# Dietary carotenoids to improve hypertension

**DOI:** 10.1016/j.heliyon.2023.e19399

**Published:** 2023-08-25

**Authors:** Firoozeh Abbasian, Mohaddeseh Sadat Alavi, Ali Roohbakhsh

**Affiliations:** a Department of Pharmacodynamics and Toxicology, School of Pharmacy, Mashhad University of Medical Sciences, Mashhad, Iran; b Pharmacological Research Center of Medicinal Plants, Mashhad University of Medical Sciences, Mashhad, Iran; c Pharmaceutical Research Center, Pharmaceutical Technology Institute, Mashhad University of Medical Sciences, Mashhad, Iran

**Keywords:** Carotenoids, Hypertension, Blood pressure, Vasodilation

## Abstract

Hypertension is one of the major risk factors for cardiovascular diseases and the main reason for premature death in older adults. Although antihypertensive medications have been used frequently, hypertension prevalence has increased in the last decade. Lifestyle improvement is a cornerstone of hypertension prevention and control. High dietary consumptions of fruits and vegetables are linked to reduced risks of high blood pressure.

Carotenoids are natural tetraterpene pigments produced by bacteria, fungi, algae, some animals, and various plants. Because of their high pharmacological potential and safety, they have been mentioned as unique therapeutic agents for a diverse range of diseases. Carotenoids modulate high blood pressure. They also have several additional benefits for the cardiovascular system, including antioxidative, anti-inflammatory, anti-atherogenic, and antiplatelet effects. They improve endothelial function and metabolic profile, as well.

In the present article, we reviewed the literature data regarding carotenoids’ influence on hypertension in both preclinical and clinical studies. Furthermore, we reviewed the underlying mechanisms associated with antihypertensive properties derived from *in vitro* and *in vivo* studies. Suppressing reactive oxygen species (ROS) production, Inhibiting angiotensin-II, endothelin-1, and oxidized low-density lipoprotein; and also nitric oxide enhancement are some of the mechanisms by which they lower blood pressure.

The present article indicated that astaxanthine, β-carotene, bixin, capsanthin, lutein, crocin, and lycopene have antihypertensive properties. Having significant antioxidant properties, they can decrease high blood pressure and concomitant comorbidities.

## Graphical abstract

**Figure:**
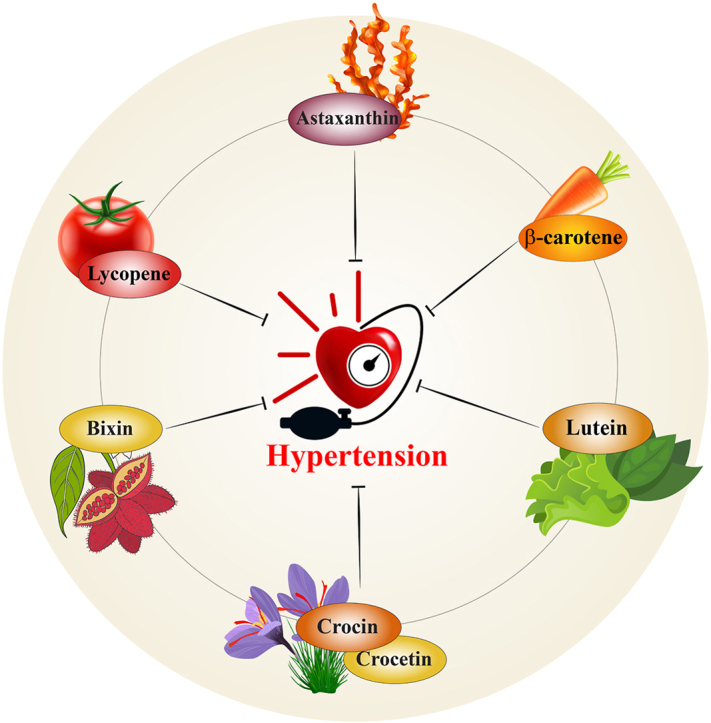

## 1 Introduction

Hypertension or high blood pressure (HBP) is one of the most prevalent chronic conditions affecting more than 1 billion people globally [1]. HBP is blamed for 9.4 million deaths per year [2]. During the last decades, numerous studies have linked hypertension with a greater risk of heart, brain, and kidney dysfunction [3]. Elevated BP changes the structure of the vessel walls in both large and small arteries and exacerbates the atherosclerotic process [4,5]. There are several pharmacological treatments available for high blood pressure. However, in some cases, medications fail to reduce blood pressure due to compensatory responses within the body or cause significant adverse reactions [6]. Dietary sodium restriction, reducing body weight, reducing stress, exercising regularly, and other modifications to dietary habits are the most effective strategies for reducing HBP [7,8].

**Fig. 1:**
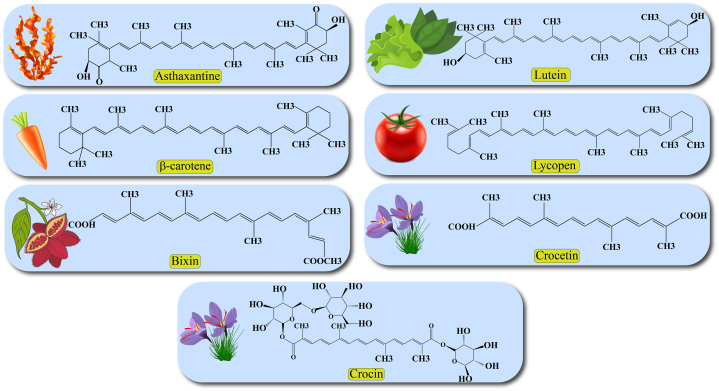
Chemical structures of carotenoids.

Carotenoids are natural red, orange, or yellow pigments that are found in plants, fruits, algae, fungi, photosynthetic bacteria, and several species of archaea. Human cells cannot produce carotenoids *de novo* and receive them from food [9]. Carotenoids contain a polyisoprenoid (tetraterpene) structure and are classified into carotenes and xanthophylls according to the presence of oxygen in their structure (Fig. 1) [10]. Carotenes, such as *α*-carotene, *β*-carotene, and lycopene contain only a parent hydrocarbon chain without any functional oxygen group. Xanthophylls, including lutein, zeaxanthin, canthaxanthin, and astaxanthin are oxygenated compounds that have oxygen atoms as hydroxy, aldehyde, carboxylic, and epoxide groups in their structure [7].

Scientific evidence suggests that carotenoids may prevent many diseases, including diabetes, osteoporosis, cancer, and particularly cardiovascular disease [11]. They are potent antioxidants that, via suppression of redox status imbalance and quenching reactive oxygen species (ROS), improve vascular function [[12], [13], [14]].

Previous investigations have provided conflicting results about the role of carotenoid supplementation or carotenoid-containing foods in regulating BP. Several studies confirmed that dietary supplementation with carotenoids significantly reduced BP [15,16], while others failed to show such an effect [17,18]. Given the above findings, we aimed to review the literature to show the putative potentials of carotenoids against hypertension in preclinical and clinical studies. We also discussed the underlying molecular mechanisms.

## 2 Methods

In this review, the following keywords including ‘carotenoid’, ‘astaxanthin’, ‘lycopene’, ‘β-carotene’, ‘lutein’, ‘crocin’, ‘crocetin’, ‘hypertension’, ‘blood pressure’, ‘vasodilation’, were used for search in Web of Science, PubMed, Scopus, and Google Scholar databases. No timelimitation was considered in this review. In the primary search, 121 studies were included. After that, duplicate researches were removed. We excluded non-English publications, conference abstracts, and dissertations. Screening of the remaining researches was performed by reading the titles, abstracts, or full texts based on the inclusion criteria. A total of 102 articles were reviewed. In order to convert an animal dose to a human dose, the human equivalent dose (HED) was used. HED was calculated using a *km* value based on the animal's weight [19,20].

### 2.1 Astaxanthin

Astaxanthin (ASX, 3,3-dihydroxy-β,β-carotene-4,4-dione) is a xanthophyll carotenoid with the molecular formula C_40_H_52_O_4_ and molar mass of 596.84 g/mol [21]. It is a lipophilic carotenoid found in algae, fish, and crustaceans [7]. Accumulating data has confirmed several biological activities for ASX, including antioxidant, anticancer, anti-inflammatory, immunomodulatory, anti-diabetic, and anti-hyperlipidemic effects [[21], [22], [23], [24]]. This dark red carotenoid is currently used as a food additive [25]. It is 5–15 times more potent than other carotenoids in terms of antioxidant properties. Considering the potent free radicals scavenging activity of ASX [26], its role in BP management has been investigated in various studies (Table 1). Hussein and co-workers investigated the antihypertensive potential of ASX in spontaneously hypertensive rats (SHRs) as a widely used model for studying the pathophysiology and management of hypertension [26]. They showed that ASX supplementation induced a significant antihypertensive effect by lowering the systolic and diastolic BP in a dose- and time-dependent manner [26]. In another study, an ASX-enriched diet reduced BP-related values, cardiovascular remodeling, and oxidative stress in SHRs [27]. ASX improved preeclamptic symptoms including the elevated BP in a rat preeclamptic model through oxidative stress suppression by reducing malondialdehyde (MDA, a marker of lipid peroxidation) content and increasing superoxide dismutase (SOD) activity [28]. In addition, oral administration of ASX considerably decreased plasma levels of oxidative parameters including nitric oxide end products such as nitrite/nitrate (NO_2_^−^/NO_3_^−^) and MDA in SHRs [29]. ASX supplementation notably declined coronary artery wall stiffness, decreased aortic wall thickness and fibrosis, and reduced the level of ROS in tunica media of hypertensive rats suggesting that ASX ameliorated hypertension-induced vascular remodeling [[29], [30], [31]]. In line with *in vivo* findings, ASX also attenuated vascular remodeling by preventing proliferation, recovering mitochondrial function, and reducing ROS in the cultured vascular smooth muscle cells (VSMC) exposed to angiotensin II [31]. Inhibition of mitochondrial fission by reducing the phosphorylation of Drp1 and increasing mitochondrial autophagy and biosynthesis via overexpression of PTEN-induced kinase (PINK), parkinson juvenile disease protein 2 (parkin), mitochondrial transcription factor A (TFAM), and peroxisome proliferator-activated receptor gamma coactivator (PGC)-1*α* are ASX underlying mechanisms [31]. It is worth to mention that oxidative stress promotes abnormal platelet function [32]. Dysfunctional endothelium-dependent vasodilatation is an important factor in the development of ischemic injuries. Hussein et al. showed that ASX delayed the prevalence of stroke in stroke-prone hypertensive rats. They suggested that ASX induced a sympatholytic effect which mediated its anti-hypertension property and protected the brain against stroke and ischemic insults. ASX also exhibited notable neuroprotective effects in ischemic mice, probably due to its antioxidant activity [26]. Another study showed that ASX mitigated elevated systolic blood pressure in stroke-prone SHRs [33]. In theses animals, thrombogenesis, also was induced using a helium-neon laser technique in pial blood vessels. ASX also protected animals from vascular oxidative damage as indicated by decreasing 8-hydroxy-2′-deoxyguanosine (8-OHdG) excretion in the urine. Moreove, nitric oxide (NO) metabolites in urine increased after ASX administration [33]. The effect of ASX on NO, which plays a key role in vascular tonicity and arterial blood pressure-mediated vasorelaxation, has been explored [26,34]. Endothelial dysfunction is defined as impaired vessel relaxation. It depends on endothelial factors, such as the generation of NO by endothelial NO synthase (eNOS). NO is responsible for regulating vascular tune, which has a modulatory effect on BP and hemostasis predominantly through relaxation activity on the smooth muscle cells and inhibition of platelet aggregation [30]. Using aortic rings, it was reported that the ASX vasorelaxation effect was NO-dependent, at the low concentration, and NO-independent, at the high dose [34]. ASX improved endothelial function by improvement in NO bioavailability on resistant arteries except the aorta [27]. It also increased sodium nitroprusside (SNP)-induced vascular relaxation. SNP is an endothelium-independent vasorelaxant factor, and its influence is related to its direct effect on vascular smooth muscles via NO donation [34]. These findings were confirmed by Xuan et al*.* [28]. They showed that *NG*-nitro-l-arginine methyl ester hydrochloride (l-NAME), a NO synthase inhibitor, induced preeclamptic signs that were prevented by ASX treatment via restoring redox status imbalance and suppression of inflammatory responses [28]. In addition, ASX reduced elevated BP and enhanced insulin sensitivity in rats treated with a heavy sucrose diet [35] as well as in Zucker fatty rats [36]. Endothelial dysfunction, which can be detected by the reduction in vascular responsiveness to vasodilators, is a major contributing factor to diabetic cardiovascular complications. One of the pathways responsible for endothelial dysfunction associated with diabetes is the binding of oxidized low-density lipoprotein (ox-LDL) to its endothelial receptor, lectin-like ox-LDL receptor 1 (LOX-1). Activation of LOX-1 leads to augmented lipid peroxidation and eNOS inactivation [37]. ASX treatment significantly reversed streptozotocin-induced diabetic endothelial dysfunction by preventing LOX-1 expression and increasing eNOS expression [22]. Moreover, Hussein et al. showed that ASX reduced the constrictive effects of α-adrenergic receptor agonist (phenylephrine), angiotensin II, and xanthine/xanthine oxidase system in the thoracic aorta of the SHR [34]. In addition, the BP lowering potential of ASX has been attributed to modifications in the renin-angiotensin (RAS) and NO systems and circulating tumor necrosis factor-α (TNF-α) and monocyte chemoattractant protein-1 (MCP-1) [35,36]. ASX treatment induced a vasodilatory effect in ganglionic cell layer vessels and reduced the TNF-α content but did not change the vascular endothelial growth factors (VEGF) expression in retinal tissue during 47 days of treatment [38]. Considering the interesting pharmacological properties of ASX, CDX-085, as a novel ASX prodrug, has been developed. Its administration in a murine model of thrombosis promoted antithrombotic and vasodilatory effects by increasing blood flow of the carotid artery [39]. In addition to preclinical investigations, clinical studies reported comparable results. A randomized, double-blind clinical trial was performed for eight weeks on 44 participants with type 2 diabetes. ASX supplementation (8 mg/day) improved the lipid profile, reduced visceral body fat mass, and decreased systolic BP [40]. In another randomized, double-blind study, 12 mg of ASX for four weeks increased choroidal blood flow velocity without significant effects on patients' BP, pulse rate, and intraocular pressure (IOP) [41]. According to animal studies, the HED for ASX is 8–35 mg/kg, while in these clinical studies lower ASX doses have been used.

**Table 1:** Carotenoids effects on blood pressure in preclinical and clinical studies.

Carotenoid	Model	Treatment dose and duration	Effect	Mechanism(s)	Reference
Astaxanthin	SHR Stroke-prone SHR	50 mg/kg, p.o., 2 weeks 50 mg/kg, p.o., 5 weeks	Decreased SBP Decreased DBP Decreased MBP	Improved NO-related mechanism	[26]
	Preeclampsia induced by l-NAME in rats	5, 15, and 25 mg/kg, p.o., 18 days	Decreased SBP Decreased DBP	Decreased oxidative stress Inhibited inflammatory damages	[28]
	SHR	5 mg/kg, p.o., 7 weeks	Decreased SBP Decreased DBP Decreased MBP	Increased NO bioavailability Decreased oxidative stress	[29]
	SHR	200 mg/kg, p.o., 11 weeks	Decreased SBP Decreased DBP	Decreased oxidative stress Improved mitochondrial function	[31]
	SHR	75 and 200 mg/kg, p.o., 8 weeks	Decreased SBP	Improved endothelial function (increase NO bioavailability and decreased oxidative stress) on resistant arteries	[27]
	Stroke-prone SHR	300 and 600 mg/kg, p.o., 3 weeks	Decreased SBP	Increase NO bioavailability Decreased oxidative stress	[33]
	High-sucrose diet fed rats	25, 50, and 100 mg/kg, p.o., 2 and 8 months	Decreased SBP	Decreased renin-angiotensin system activity Improved NO system	[35]
	Hypertensive and diabetic (type 2) patients	8 mg/kg, p.o., 8 weeks	Decreased SBP No effect on DBP	–	[40]
β-carotene	SHR and stroke-prone SHR	20 mg/kg, p.o., 10 weeks	Decreased SBP	Decreased oxidative stress	[45]
	Hypertensive patients	100 μg/kg	Decreased SBP Decreased DBP		[16]
	High-carbohydrate, high-fat diet fed rats	5%, p.o., 8 weeks	Decreased SBP	No effect on oxidative stress	[54]
Bixin	High-cholesterol diet fed rats	100 mg/kg, p.o, 28 days	Decreased SBP		[56]
Lutein	Hypertension induced by l-NAME in rats	0.5 and 2 mg/kg, p.o., 3 weeks	Decreased MBP Decreased SBP Decreased DBP	Decreased oxidative stress	[61]
	Hypertensive patients	100 μg/kg	Decreased SBP Decreased DBP		[16]
Lycopene	SHR	10 mg/kg, p.o., 4 weeks	Decreased SBP	Decreased oxidative stress	[95]
	Hypertension induced by angiotensin-II in rats	10 mg/kg, p.o., 2 weeks	Decreased SBP		[97]
	Hypertensive (stage 1) patients	250 mg/kg, p.o., 8 weeks	Decreased SBP		[102]
	Overweight men/women	Tomato-enriched diet, 6 weeks	Decreased DBP		[91]
	Prehypertensive patients	7 mg/day, p.o., 4 weeks	Decreased MBP		[104]
	Hypertensive patients	15 mg/day, p.o., 6 weeks	Decreased SBP Decreased DBP		[15]
	Hypertensive patients	5, 15, 30 mg/day, p.o., 8 weeks	Decreased SBP		[110]
	Prehypertensive healthy adults	15 mg/day, p.o., 12 weeks	No effect		[18]
	Hypertensive patients with high cardiovascular disease risk	75 and 213 mg/kg of tomato extract, p.o., 4 weeks	Decreased SBP Decreased DBP Decreased mean arterial pressure		[111]
	Hypertensive (stage 1) patients	200 g tomato fruit juice, p.o., 4 weeks	No effect		[93]
	Primigravida women	4 mg/day, p.o., until delivery	Decreased mean DBP Decreased pre-eclampsia risk	Decreased oxidative stress	[105]
	Healthy overweight middle-aged adults	10 mg/day of tomato diet, 4 weeks	No effect		[94]
	Patients with cardiovascular disease	7 mg/day, p.o., 2 months	No effect		[17]
	Healthy men	6 and 15 mg/kg, p.o., 8 weeks	Decreased SBP		[112]
	Hypertensive patients	100 μg/kg	Decreased SBP Decreased DBP		[16]
Crocin	Hypertensive rats induced by desoxycorticosterone acetate	50, 100, and 200 mg/kg, 5 weeks	Decreased MBP Decreased SBP		[67]
	Hypotension induced by diazinon in rats	12.5, 25, and 50 mg/kg, p.o., 4 weeks	Restore SBP	Decreased oxidative stress	[66]
	Hypertension induced by angiotensin-II in rats	200 mg/kg, slow i.v., 2 weeks	Decreased MBP Decreased SBP	Increase antioxidant activity	[70]
	Isoproterenol treated rats	5, 10, and 20 mg/kg, p.o., 21 days	Decreased MBP Decreased SBP Decreased DBP	Decreased oxidative stress	[69]
	Metabolic syndrome‐induced osteoporosis in rats	5 and 10 mg/kg, p.o., 5 days/week, 12 weeks	Decreased MBP		[71]
	High fat diet fed rats	20 mg/kg, p.o., 49 days	Decreased SBP		[65]
	Pulmonary arterial hypertension induced by monocrotaline in rats	30 mg/kg, p.o., 21 days	Decreased mean arterial pressure	Decreased oxidative stress	[74]
	Central retinal artery occlusion in rabbits' eyes	10 mg/kg, i.p., 30 min after injury	Reduced intraocular pressure	Decreased oxidative stress Increase NO bioavailability	[78]
	Diabetic (type 2) patients	30 mg, p.o., 12 weeks	Decreased SBP		[80]
Crocetin	High fructose diet fed rats	20, 40 mg/kg, p.o., 8 weeks	Decreased SBP	Prevented adiponectin expression Inhibited inflammatory response	[82]
	Stroke-prone SHR	25 and 50 mg/kg, p.o., 3 weeks	Decreased SBP	Increased NO bioavailability Decreased oxidative stress	[83]
	Hemorrhagic shock induced by cardiac injury in rats	50 mg/kg 40 min after bleeding	Increased mean arterial pressure	Prevented inflammatory cascades Suppressed oxidative stress	[88]

DBP: Diastolic blood pressure, SBP: Systolic blood pressure, SHR: Spontaneously hypertensive rats, MBP: Mean blood pressure, NO: Nitric oxide.

### 2.2 β-carotene

β-carotene is generally considered a precursor of vitamin A. It is an intensely colored red-orange pigment with the C_40_H_56_ formula and a molecular weight of 536.8726 g/mol. It is abundant in fungi, plants, and fruits like carrots and apricots.

In several randomized controlled trials, β-carotene supplementation reduced different cancer incidences including pancreatic, colorectal, prostate, and breast cancers [42]. Additionally, it prevents skin damage caused by photooxidative stress [43].

The effects of β-carotene on the cardiovascular events and HBP have been assessed in both preclinical and clinical studies. β-carotene supplementation decreased atherosclerosis in cholesterol-fed rabbits [44]. It preserved endothelium-dependent vessel relaxation via vascular tissue antioxidant content augmentation [44]. Another study showed the significant hypotensive influence of β-carotene supplementation in SHRs and stroke-prone SHRs [45]. The hypotensive mechanism of β-carotene has been attributed to its antioxidant properties since it significantly reduced serum levels of MDA. Although β-carotene significantly decreased BP, it could not inhibit elastosis in the SHR carotid arteries [45]. It was indicated that β-carotene reduced elevated BP in diabetic SHRs. It also reduced oxidative stress in the retina by decreasing the level of superoxide anion, 8-OHdG, and nicotinamide adenine dinucleotide phosphate (NADPH) oxidase [46]. In line with animal findings, in patients suffering from both hypertension and diabetes mellitus, arterial stiffness and lipid peroxidation increase gradually, concurrently with reduced plasma levels of β-carotene [47]. Fazal et al*.* evaluated the impact of β-carotene on the expression level of the angiotensin-converting enzyme (*ACE*) gene, responsible for thioacetamide-induced rat renal toxicity [48]. Renal *ACE* gene expression enhancement raised BP and increased hypertensive end-organ renal damage [49]. The rats receiving β-carotene showed diminished expression of the *ACE* gene. β-carotene also protected renal tissues against thioacetamide-induced oxidative damage by reducing lipid peroxidation and antioxidant enzymes enhancement [48]. Dietary supplementation of β-carotene potentially increased antioxidant enzyme activities and enhanced NO production by regulating gut microbiota in sows [50]. Epidemiological studies indicated that higher circulating levels of α-carotene or β-carotene are linked to decreased risk of hypertension and atherosclerosis in young and elderly patients [5,[51], [52], [53]]. Also, daily supplementation with f α-carotene and β-carotene in hypertensive patients reduced hypertension risk [16]. In a study by Poudyal et al. a high-carbohydrate, high-fat diet developed hypertension, cardiac fibrosis, hyperlipidemia, and insulin resistance as well as increased plasma markers of oxidative stress and inflammation in rats. Insulin resistance contributes causally to hypertension pathogenesis. β-carotene did not modify these changes, while purple carrot juice restored [54]. The recommended dose of α-carotene and β-carotene in hypertensive patients is about 100 μg/kg/day [16].

### 2.3 Bixin

Bixin, also known as annatto, is widely used as a colorant in different foods such as bakery products, vegetable oils, beverages, and dietary supplements [55]. It is a natural orange-red colored achiote pigment with the molecular formula C_25_H_30_O_4_ and a molecular weight of 394.5 g/mol. Only one study investigated its role in the cardiovascular system. Bixin treatment showed a significant reduction in BP of animals fed a high-cholesterol diet [56]. Using the results of this study, we calculated an HED of 16 mg/kg.

### 2.4 Lutein

Lutein (β, ε-carotene- 3, 3-diol), with a molecular formula of C_40_H_56_O_2_ and a molecular weight of 568.871 g/mol, is a red-orange carotenoid mainly extracted from marigolds. It is the main constituent of the macular pigment, along with its isomer zeaxanthin. Lutein prevents age-related macular degeneration and cataracts, reducing the risk of stroke, cardiovascular diseases, and cancer [57].

Armoza et al. investigated the effect of lutein on two cultured endothelial cell models, including EA.hy926 and human umbilical vein endothelial cells (HUVEC) [58]. Lutein significantly enhanced basic endothelial function as presented by amplified NO, declined endothelin (ET-1) release, and attenuated inflammatory mediators release [58]. In the hyperhomocysteinemia-induced endothelial impairment model in rats, similar findings were reported [59]. Lutein treatment reversed NO reduction and increased ET-1, and enhanced the expression of nuclear factor (NF)-κB p65 and intercellular adhesion molecule (ICAM)-1 as pro-inflammatory makers [59].

In response to various stimuli, including ROS, inflammatory mediators, mechanical forces, and catecholamines, endothelial cells release chemokines, and adhesion molecules, such as ICAM-1 and vascular cell adhesion molecule (VCAM)-1. Angiotensin II-caused hypertension was linked to overexpression of vascular ICAM-1, and that was attenuated by NADPH oxidase inhibition [60].

Lutein's antihypertensive potential on l-NAME-caused hypertension in rats was also assessed. Lutein significantly reduced mean, systolic, and diastolic BP and inhibited heart rate reduction induced by l-NAME. Moreover, l-NAME also caused cardiac hypertrophy and increased kidney mass index, which were prevented by lutein. l-NAME also increased plasma MDA levels and caused severe depletion of plasma GSH. Lutein restored plasma nitrite levels and diminished oxidative stress in a concentration-dependent manner [61]. In a clinical trial study, lutein supplementation (20 mg/kg, eight weeks) reduced inflammatory cytokines in early atherosclerosis patients [62]. Li and colleagues in their epidemiological study found that daily lutein administration (100 μg/kg) significantly decreased the risk of hypertension in adults [16].

### 2.5 Crocin

Crocin (a diester of the disaccharide gentiobiose, C_44_H_64_O_24_, 976.96 g/mol) is the red-colored component of saffron that easily dissolves in water [63]. A significant body of evidence has confirmed that crocin exhibits strong antioxidant, anti-inflammatory, and anti-tumor effects [64]. Several studies have shown that the cardioprotective properties of crocin are related to its BP-lowering effects. In an animal study, supplementation for 49 days with a high fat diet elevated mean arterial, systolic, and diastolic BP and increased heart rate. Crocin treatment showed hypotensive effects especially on systolic BP (Table 1) [65]. In the study by Razavi et al*.,* crocin, *per se,* did not affect systolic BP and heart rate [66]. Chronic administration of crocin decreased mean and systolic BP in desoxycorticosterone acetate (DOCA)-salt hypertensive rats, dose-dependently. The researchers showed that crocin did not change BP and heart rate in normotensive rats [67]. They also compared crocin and safranal's hypotensive potential. The findings indicated that safranal was more effective than crocin for HBP control [68]. Isoproterenol-treated rats displayed cardiac dysfunction by lowering systolic, diastolic, and mean arterial BP. Crocin significantly modulated hemodynamics and showed cardioprotective effects in isoproterenol-treated rats through modulation of oxidative stress [69]. The effect of crocin on cardiovascular responses in rats with acute hypertension caused by angiotensin II was further investigated. Angiotensin II increased mean arterial, systolic BP, and heart rate, which were reversed by crocin pretreatment [70]. The researchers believed that the hypotensive effects of crocin at the highest dose were similar to losartan and were mediated by its antioxidant activity [70]. Crocin also suppressed elevated mean arterial BP in a rat model of metabolic syndrome‐induced osteoporosis. It also reduced oxidative stress and suppressed inflammatory mediators in bone tissues [71].

Gestational hypertension is one of the complications of pregnancy induced by preeclampsia, which may cause prenatal and maternal mortality and morbidity [72]. Crocin exhibited an antihypertensive effect in a rat model of preeclampsia induced by l-NAME [73]. It alleviated inflammation and redox imbalances in the serum and placental tissues. This ameliorated pregnancy outcomes in terms of fetal survival, fetal weight, and the fetal/placental weight ratio. Crocin stimulates the placental nuclear factor-erythroid 2-like 2 (Nrf2)/heme oxygenase-1 (HO-1) pathway. Nrf2 and HO-1 are transcription factors that contribute to the cellular response to oxidative stress and cytokines release, thus protecting tissues against redox status [73]. Oxidation resistance 1 (OXR1) acts as an essential sensor of cellular oxidative stress by stimulating P21 and Nrf2 upregulation. In a rat model of pulmonary arterial hypertension (PAH) induced by a single dose of monocrotaline, OXR1 and P21 gene expression and antioxidant capacity in lung tissue significantly decreased. Treatment with Crocin significantly promoted hemodynamic and oxidative stress biomarkers in PAH rats. This was associated with enhanced levels of OXR1 and downstream target genes [74].

In another study to investigate the mechanism(s) underlying crocin's vasodilatory effects, isolated rat thoracic aorta rings were contracted by phenylephrine or KCl [75]. Crocin caused relaxation in rings precontracted with phenylephrine in a dose-dependent manner but not in KCl-precontracted aortic rings. As the relaxant potential of crocin was abolished by incubating aortic rings with l-NAME, the vasodilatory effect of crocin was endothelium-dependent and mediated via NO production [75]. It also exhibited potent anti-contractile effects against phenylephrine in aortic rings. Crocin improved endothelial function through stimulation of the eNOS/NO pathway in both HUVECs and human umbilical artery endothelial cells (HUAECs). This functional improvement was associated with intermediate-conductance Ca^2+^-activated K^+^ channels (KCa3.1) via the mitogen-activated protein kinase/extracellular-signal-regulated kinase (MEK/ERK) and phosphoinositide 3-kinases (PI3K), serine/threonine protein kinase B (PKB; also known as Akt) signaling pathway activation [76]. The inhibitory effects on extracellular Ca^2+^ influx by the endoplasmic reticulum might be the underlying mechanism of crocin-induced vasodilation [77]. Crocin's beneficial effect on the eye has been reported. It increased blood flow in the retina and choroid and improved retinal function recovery following central retinal artery occlusion in rat eyes. It was suggested that crocin increased blood flow due to vasodilation, and subsequently improved oxygenation and nutrient supply to retinal structures [78].

Hypertension has been associated with hyperinsulinemia and insulin resistance in humans [79]. In a randomized clinical trial, 3 months supplementation with 30 mg of crocin significantly improved glycemic control and insulin resistance. It also decreased systolic BP in patients with type 2 diabetes [80]. As a result, crocin's HED is between 10 and 30 mg/kg.

### 2.5 Crocetin

Crocetin is a deglycosylated derivative of crocin with a C_20_H_24_O_4_ formula and 328.402 g/mol molecular weight [63]. In addition to saffron, it is found in gardenia fruits. Crocetin exhibits biological activity profiles such as anti-tumor, neuroprotection, anti-diabetics, anti-inflammatory, antiatherosclerotic, and anti-hyperlipidemic effects. Improvement of oxygenation in hypoxic tissues, antioxidant effects, suppression of pro-inflammatory cytokines release, and anti-proliferative activity are selected underlying mechanisms of its protective effects [81].

High fructose consumption results in pathological changes such as insulin resistance, dyslipidemia, and hypertension in rats resembling metabolic syndrome. Crocetin administration effectively controlled high systolic BP and down-regulated the expression of both protein and mRNA of adiponectin and TNF-α [82]. The protective effects of crocetin were evaluated against hypertension and cerebral thrombosis in stroke-prone SHRs. Crocetin induced antihypertensive (decreased systolic BP) and antithrombotic effects related to increased NO bioavailability, probably by decreased NO inactivation [83]. Furthermore, the brain tissue of stroke-prone hypertensive rats exhibited excessive amounts of ROS. A high dose of crocetin decreased redox status imbalance in stroke-prone hypertensive rats’ brains [84]. Tang et al. evaluated endothelial dysfunction induced by a high cholesterol diet in rabbits and by treating bovine aortic endothelial cells (BAECs) with ox-LDL. They measured the endothelium-dependent relaxation evoked by acetylcholine and endothelium-independent relaxation mediated by SNP in the thoracic aorta isolated from rabbits. Crocetin dose-dependently improved endothelium-dependent relaxation and restored maximal relaxation with no effect on endothelium-independent relaxation. Crocetin simultaneously increased serum NO levels, upregulated vessel mRNA expression of eNOS, and vessel cyclic GMP (cGMP) content. INOS activity remained unchanged. In BAECs, ox-LDL treatment decreased NO production and down-regulated eNOS activity and mRNA expression, which were reversed following crocetin dose-dependently [85]. Llorens and co-workers investigated the role of NO and prostanoids in the endothelial modulation of crocetin in SHRs. Crocetin diminished the maximum contractility of phenylephrine that was prevented by l-NAME, but not by indomethacin treatment [86].

Similarly, crocetin enhanced acetylcholine relaxation in hypertensive rats' aorta but not in normotensive rats. Similar to the study mentioned previously, l-NAME but not indomethacin treatment prevented relaxant response in hypertensive rats. These results recommend that crocetin exerts vasomodulatory effects in hypertension by improving endothelium-dependent relaxation via endothelial NO but not the cyclooxygenase pathway [87].

Yan et al. investigated the effects of crocetin on cardiac injury produced by hemorrhagic shock and resuscitation in rats. They administered crocetin via the duodenum at 50 mg/kg and 40 min after bleeding and found that mean arterial pressure increased. Moreover, serum creatine kinase activity, iNOS, TNF-α, IL-6, and NF-ĸB elevation induced by hemorrhagic shock were reversed. Furthermore, they found that crocetin inhibited ROS generation and increased SOD activity to block inflammation [88]. Crocetin's HED is estimated to be between 3 and 8 mg/kg.

### 2.6 Lycopene

**Fig. 2:**
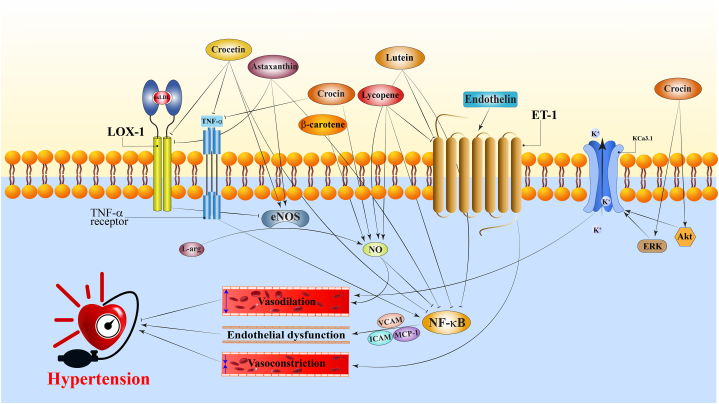
A schematic illustration of antihypertensive mechanisms of carotenoids. Carotenoids by inhibition of ROS, angiotensin II, and NF-κB induce antihypertensive effects. → present the promote/activate and ⊥ present the inhibitory/suppressive effects. CAT: catalase, eNOS: endothelial nitric oxide synthase, GR: glutathione reductase, GSH-Px: glutathione peroxidase, HO-1: heme oxygenase-1, L-arg: l-arginine, LOX-1: lectin-like ox-LDL receptor 1, NADPH oxidase: nicotinamide adenine dinucleotide phosphate oxid, NF-κB: nuclear factor-κB, NO: nitric oxide, Nrf2: nuclear factor-erythroid 2-like 2, ox-LDL: oxidized low-density lipoprotein, ROS: reactive oxygen species, SOD: superoxide dismutase.

**Fig. 3:**
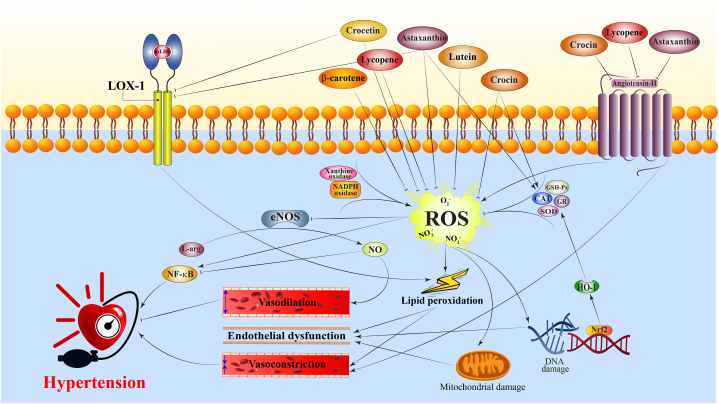
A schematic illustration of antihypertensive mechanisms of carotenoids. Carotenoids by inhibition of inflammation (TNF-α, LOX-1, NF-κB, VCAM, ICAM, and MCP-1), endothelin-1 receptor, activation of KCa3.1 potassium channels, and intensifying NO production induce antihypertensive effects. → present the promote/activate and ⊥ present the inhibitory/suppressive effects. Akt: protein kinase B, eNOS: endothelial nitric oxide synthase, ERK: extracellular signal-regulated kinase, ET-1: endothelin-1 receptor, ICAM: intercellular adhesion molecule, L-arg: l-arginine, LOX-1: lectin-like ox-LDL receptor 1, NO: nitric oxide, MCP-1: monocyte chemoattractant protein-1, ox-LDL: oxidized low-density lipoprotein, TNF-α: tumor necrosis factor-α, VCAM: vascular cell adhesion molecule.

Lycopene (C_40_H_56_, 536.873 g/mol) is a fat-soluble, unsaturated carotenoid found in red-colored fruits and vegetables, including tomatoes, watermelon, papaya, red grapefruit, and guava. Lycopene's pharmacological effects include antiatherosclerotic, antioxidant, anti-inflammatory, antihypertensive, cardioprotective, antiplatelet, anti-atherogenic, anti-apoptotic, and improving endothelial function [89,90]. In several randomized clinical trials, a tomato-enriched diet could significantly decrease BP in overweight men/women [91], healthy male subjects who received fat meals [92], and people suffering from uncontrolled hypertension [15]. However, there are reports showing that tomato extract was not able to reduce HBP [18,93,94]. Cardiovascular complications such as endothelial dysfunction, cardiac remodeling, renal alteration, and oxidative stress are associated with hypertension, and these complications worsen with age. There was an effective reduction in systolic BP in both young and aged rats [95]. Lycopene treatment also improved age-associated harmful changes in hypertensive rats, such as cardiac and renal remodeling and oxidant-antioxidant imbalance [95]. Moreover, lycopene inhibits ACE activity and attenuates angiotensin–II–induced oxidative stress (Fig. 2) [96]. Angiotensin-II enhanced HBP, and a lycopene-enriched diet attenuated hypertension development and ameliorated cardiovascular remodeling and oxidative stress in hypertensive rats [97]. Combination of tomato extract with low doses of ACE inhibition, calcium channel blockers, or their combination with low dose diuretics for six weeks reduced BP by more than ten mmHg systolic and more than five mmHg diastolic pressure [15]. In addition, lycopene reduced vascular superoxide anion and lipid peroxidation and modified antioxidant enzyme activity impaired by angiotensin-II [97]. Similar to previous studies, lycopene improved endothelial function as measured by improved NO and reduced ET-1 release. It also attenuated inflammatory NF-κB signaling by decrement of TNF-α-induced leukocytes adhesion, prevention of ICAM-1 and VCAM-1 expressions, and suppression of NF-κB components nuclear translocation (Fig. 3) [98]. Blockade of the VCAM-1 ligand with monoclonal antibodies decreased atherosclerosis in apoE-deficient mice [99].

Previous clinical studies have documented the role of lycopene on blood pressure with contradictory results [100,101]. A double-blind, placebo-controlled study showed that 8 weeks of treatment with natural antioxidants from tomato extract (lycopene, β-carotene, phytoene, phytofluene, vitamin E, phospholipids, phytosterol) reduced systolic BP in patients with grade-1 hypertension [102]. Lycopene reduces hypertension in overweight/obese people [103]. Based on the National Health and Nutrition Examination Survey (NHANES(, 2001–2006, researchers examined the relations between serum uric acid, serum lycopene, and hypertension. It was found that there was a significant positive link between serum uric acid and hypertension. In contrast, there was a significant inverse association between serum lycopene and hypertension. Furthermore, there was a significant association between the ratio of serum lycopene and the lower level of hypertension in adults who are overweight/obese [103]. In prehypertension people (systolic BP 130–139 mmHg, and diastolic BP 80–89 mmHg), supplementation with a formulation of dark chocolate and lycopene for 4 weeks attenuated systemic BP and serum lipids [104]. In addition, preeclampsia prevalence in primigravida women treated with lycopene had a lower rate than in untreated control subjects [105]. However, supplementation with lycopene in people suffering from cardiovascular disease could not change HBP and arterial stiffness compared with the placebo group [17]. Li et al. performed analysis based on NHANES, 2007–2014, to evaluate the association between lycopene intake from diet and supplements with hypertension. They recommended that a daily intake of 100 μg/kg/day of lycopene in adults is significantly associated with reduced hypertension risk [16].

## 3 Concluding remarks

Various strategies can be used to treat hypertension, including lowering the heart rate and cardiac output, dilation of arteries, reducing blood volume, and inhibiting the sympathetic nervous system. An unhealthy diet is associated with many pathological conditions, including hypertension and other cardiovascular diseases. Additionally, atherosclerosis increases the risk of hypertension and stroke, which are associated with a significant increase in morbidity and mortality. In this review article, studies about some carotenoids and their effects on hypertension and the cardiovascular system were summarised. A long list of studies showed that carotenoids reduce hypertension in humans and animals (Table 1).

According to data from NHANES epidemiological survey, serum levels of α- and β-carotene, lutein and lycopene significantly reduced the risk of hypertension in the general adult population [106]. In other words, dietary intake of α- and β-carotene, lycopene, lutein and zeaxanthin at 100 μg/kg/day was reversely correlated with hypertension risk [16]. A recent prospective study investigated the association between serum carotenoids concentrations and mortality risk in hypertensive adults. The results indicated that low serum levels of β-carotene, lycopene, and lutein/zeaxanthin were associated with a higher risk of all-cause and cardiovascular mortality in hypertensive adults [107].

Many molecular targets have been associated with carotenoids' beneficial effects on HBP. As shown in Fig. 2, Fig. 3, the main mechanisms are increasing NO system's activity and antioxidant properties. Evidence from previous studies establishes a strong link between redox status, inflammation, and hypertension [4,108,109].

Several factors in the hypertensive location, such as angiotensin II, sodium, and catecholamines increase ROS generation. NADPH oxidase is a major enzyme complex involved in this response, but mitochondria also produce excessive ROS levels under hypertension. Uncoupled nitric oxide synthase and xanthine oxidase have also been implicated in ROS formation during hypertension. These changes are promoted in endothelial, vascular smooth muscle, neuronal, and renal tubular cells. Thus, oxidative stress causes endothelial dysfunction, cardiovascular remodeling, vascular damage, and sympathetic system activation. In addition, infiltrating macrophages augment local ROS levels. A major effect of ROS is the promotion of inflammation, in part by activating redox sensitive transcription factors specially NF-κB. In hypertensive patients, NO bioavailability is reduced which normally inhibits NF-κB via several mechanisms. Local inflammation is a major player in end-organ damage in hypertension.

Carotenoids improve atherosclerosis by improving NO availability, endothelium-dependent vasodilation improvement, and reduction in protein, lipid, DNA, and mitochondrial damages. Carotenoids reduce factors that activate pro-oxidant enzymes, such as NADPH oxidase, inflammatory cytokines and angiotensin II. In addition, carotenoids prevent the overexpression of IL-6, IL-1*β*, TNF-*α*, ICAM-1, and VCAM-1 during inflammation. Cohort studies show plasma carotenoids levels are lowered immediately after an ischemic stroke. Carotenoids intake including lycopene, α- and β-carotene, lutein, and zeaxanthin reduces the risk of stroke-related mortality and morbidity.

Another mechanism of HBP reduction by carotenoids is interaction with angiotensin II. For example, the blood pressure-lowering effect of crocin is comparable to the well-known effect of losartan which decreases blood pressure by antagonizing the angiotensin II receptor. Moreover, *ACE* gene expression was diminished in animals receiving β-carotene. Carotenoids have been shown to have antihypertensive effects in most basic studies, however some clinical studies indicate they could not affect HBP. Taken together, it might be suggested that carotenoid consumption during life may delay hypertension development and may decrease high blood pressure. They may also reduce comorbid conditions due to their antioxidant and anti-inflammatory properties.

## Author contribution statement

All authors listed have significantly contributed to the development and the writing of this article.

## Data availability statement

No data was used for the research described in the article.

## Declaration of competing interest

The authors declare that they have no known competing financial interests or personal relationships that could have appeared to influence the work reported in this paper.
